# DMSO and TMAO—Differences in Interactions in Aqueous Solutions of the K-Peptide

**DOI:** 10.3390/ijms23031872

**Published:** 2022-02-07

**Authors:** Julia Godlewska, Bartosz Cieśla, Jarosław Wawer, Piotr Bruździak

**Affiliations:** 1 Department of Physical Chemistry, Gdańsk University of Technology, Narutowicza 11/12, 80-233 Gdańsk, Poland; s180776@student.pg.edu.pl (J.G.); jaroslaw.wawer@pg.edu.pl (J.W.); 2 Department of Inorganic Chemistry, Gdańsk University of Technology, Narutowicza 11/12, 80-233 Gdańsk, Poland; s180816@student.pg.edu.pl

**Keywords:** biomolecular interactions, amyloid, intermolecular interactions, protein—ligand binding, chaperones, microcalorimetry, spectroscopic techniques, DFT calculations

## Abstract

Interactions between a solvent and their co-solute molecules in solutions of peptides are crucial for their stability and structure. The K-peptide is a synthetic fragment of a larger hen egg white lysozyme protein that is believed to be able to aggregate into amyloid structures. In this study, a complex experimental and theoretical approach is applied to study systems comprising the peptide, water, and two co-solutes: trimethylamide *N*-oxide (TMAO) or dimethyl sulfoxide (DMSO). Information about their interactions in solutions and on the stability of the K-peptide was obtained by FTIR spectroscopy and differential scanning microcalorimetry. The IR spectra of various osmolyte–water–model-peptide complexes were simulated with the DFT method (B3LYP/6-311++G(d,p)). The FTIR results indicate that both solutes are neutral for the K-peptide in solution. Both co-solutes affect the peptide to different degrees, as seen in the shape of its amide I band, and have different influences on its thermal stability. DFT calculations helped simplify the experimental data for easier interpretation.

## 1. Introduction

Protein or peptide aggregation is a natural phenomenon that, in certain circumstances, can be dangerous. One such deleterious process is the formation of amyloid fibrils. Amyloids are large protein aggregates that are resistant to many degradation mechanisms that arise as a result of improper folding. The structure of amyloids is rich in β-sheets [1], and the formed aggregates are unbranched and long and have diameters in the range of nanometers (2–12 nm) [2,3]. Around 40 diseases are believed to be linked to the formation of amyloids [4], including Alzheimer’s disease, Parkinson’s disease, and type II diabetes.

The role of such protein assemblies in pathogenesis is unknown or even questioned. Oligomers have been suggested to potentially be more toxic than mature fibrils [5,6]. The biochemical systems involved in fibrillation are complex, and several possible routes have been identified in the regulation of this process [7]. However, the amyloidogenic potency of the protein itself is one of the key factors that undoubtedly plays an important role in this process. Some amino-acid sequences have been shown to have a greater ability to form amyloids [8]. The role of aromatic AA residues is especially important [9]. Keeping all of this in mind, great effort have been undertaken to identify the core part of a protein that triggers amyloidogenesis [10,11,12].

Due to the slow kinetics of amyloidogenesis and other difficulties related to in vitro studies, the exact mechanism of in vivo aggregation is still under debate. Moreover, such a vague connection between aggregation and pathogenicity makes developing a treatment method more difficult. Thus, studies on amyloid formation are an emerging direction of modern biochemistry. Research on the properties of model systems is particularly helpful in this matter.

Hen egg white lysozyme (HEWL) is one of the most extensively investigated model proteins for which fibrillation analyses have been undertaken. A coarse estimation of the core amyloidogenic sequence of HEWL was performed in the early 2000s [13,14]. Almost a decade later, the position of this region was further determined with higher precision by Sugimoto et al. [15,16]. This fragment consists of residues 54–62 GILQINSRW and was named the K-peptide. The effect of fibrillation conditions on HEWL amyloidogenesis was tested on many occasions. Among others, the influences of temperature and pH [17], fibrillation in the presence of low molecular additives [18,19,20], impact of ionic strength [21], and shear or stirring rate [22,23] were examined. Investigations on peptide fibrillation have been much more limited. However, certain issues still remain unexplored. For example, dimethyl sulfoxide (DMSO) is frequently used to improve the solubility of the substrate [16] or investigated additives [24,25], but examinations on how this solvent influences amyloidogenesis have been rarely conducted [23].

In this work, we attempt to answer whether DMSO can interact with the K-peptide in a solution. Then, our findings are compared with those for another similar system comprising trimethylamine *N*-oxide (TMAO), for which the stabilizing properties are well known and described [26,27,28,29]. Both compounds are important chemical chaperons widely used to improve the thermal- and cryo-stabilization, aggregation, folding, etc. of macromolecules. Although similar in size and overall structure, they act differently and affect solvent media in different manners [30,31]. Some protocols focus on the measurement and isolation of these osmolytes’ spectra. Such spectra are easier to analyze and less convoluted than the FTIR spectra of peptides but provide the same information about possible osmoyte–peptide interactions. The role of molecular interactions, elucidated with the aid of our studies, may provide deeper insights into the mechanism of amyloid formation.

## 2. Materials and Methods

All solutions of TMAO (≥99.0%, Sigma-Aldrich, Darmstadt, Germany), DMSO (99.9+%, Alfa Aesar, ThermoFisher GmbH, Kandel, Germany), and the K-peptide (98%, Lipopharm.pl, Zblewo, Poland) were prepared by weight using a XS205 DualRange analytical balance (Mettler Toledo, Greifensee, Switzerland). The densities of DMSO and TMAO solutions at 25.000 ${}^{{}^{\circ}}$C were measured using a DMA 5000 densimeter (Anton Paar, Graz, Austria). The densities of the K-peptide solutions were approximated using the partial molar volume of a peptide equal to 0.73 cm${}^{3}$·g${}^{-1}$. The densities were necessary to calculate the proper molar concentrations (mol·dm${}^{-3}$).

### 2.1. ATR-FTIR Spectroscopy

All ATR-FTIR spectra were collected using a Nicolet 8700 FTIR spectrometer (Thermo Scientific, Waltham, MA, USA) and OMNIC software (Thermo Scientific, Waltham, MA, USA). The spectrometer was equipped with a six-reflection Ge crystal thermostated ATR accessory (Specac Ltd., Orpington, Great Britain), KBr beamsplitter, DTGS-TEC detector, and EverGlo IR source (all from Thermo Scientific, Waltham, MA, USA). A thermal circulator (Julabo GmbH, Seelbach, Germany) was used to maintain the temperature of the ATR accessory at a constant level of 25.0 ± 0.1 ${}^{{}^{\circ}}$C. Each spectrum was an average of 256 independent scans with a resolution of 2 cm${}^{-1}$. The spectrometer and the accessory were purged with dry nitrogen (NiGen LCMS 40-1 nitrogen generator, Claind, Lenno, Italy). However, any excess water vapor contribution was subtracted with the aid of our recently developed algorithm [32].

For each osmolyte (DMSO and TMAO), two kinds of solution series were prepared. The first one consisted of six solutions of a fixed molar concentration of osmolyte (DMSO: 6.7±1.2 mol·dm${}^{-3}$; TMAO: 2.02±0.08 mol·dm${}^{-3}$) and varying K-peptide concentration within the range 0.0–40.0 mg·mL${}^{-3}$. The easiest way to prepare this series is, first, to prepare six K-peptide aqueous solutions of various concentrations; next, dissolve a fixed amount of the osmolyte in a fixed volume of the K-peptide’s solution to maintain a constant molar concentration of the osmolyte. Errors during the preparation procedure are inevitable, but precise analytical balance and weighing each solution at each preparation step allowed us to minimize these errors and to calculate the molar concentrations or molalities necessary for the next steps with high confidence. This series was used to determine if any interaction between the osmolyte and K-peptide were formed. The second kind of solution series consisted of five aqueous solutions of the osmolyte, where its concentration was close to that of the first series but varied slightly in each sample (DMSO: 6.07÷4.50 mol·dm${}^{-3}$; TMAO: 2.93÷1.31 mol·dm${}^{-3}$). This series allowed us to determine the influence of concentration changes on the band shape of DMSO or TMAO spectra. Spectra of all solutions were collected 1 to 2 h after their preparation. In the case of the DMSO solutions, the K-peptide was fully soluble, while in the presence of TMAO, the K-peptide formed a suspension rather than a typical peptide/protein solution. However, the goal of these studies was to investigate the intermolecular interactions with a focus on the vibrational structure of the DMSO or TMAO molecule in such systems. All of the spectra for water were accounted for during analysis (as they have an easily recognizable difference in bands in the OH bending region 1700–1600 cm${}^{-1}$) according to its molar concentration in a sample. Next, all of the spectra were divided by the factor of $1/C_{\mathrm{DMSO}\;\mathrm{or}\;\mathrm{TMAO}}$, which allowed us to obtain the molar spectra of DMSO or TMAO in their pure solutions or solutions of the K-peptide. Such a normalization procedure is necessary to visualize the changes in spectra with the aid of a derivative spectrum and with greater confidence.

A variant of the difference spectra method was applied to extract the so-called derivative or changeability spectra from each series (sometimes called excess spectra). This name should not be confused with ordinary spectra derivatives. The derivative is defined as ${\left(\partial \epsilon /\partial m\right)}_{m=0}$, where ϵ is the molar absorption coefficient at a given wavenumber of a molar spectrum and *m* is the molality of the solution (mol·${\mathrm{kg}}_{\mathrm{water}}^{-1}$), and details of the calculation method can be found in our previous papers [33,34]. This derivative emphasizes small, sometimes obscure, variations in spectral series, i.e., shifts, or width or intensity changes in IR bands. Then, these alterations can be interpreted with the aid of DFT calculations in the context of intermolecular interactions. This kind of spectrum also serves to calculate “affected spectra”; however, in this paper, all of the information available directly from the derivative spectrum was sufficient.

### 2.2. Differential Scanning Calorimetry

All calorimetric measurements were performed using a nanoDSC III calorimeter (Calorimetry Sciences Corp., Lindon, UT, USA) equipped with platinum capillary cells (300 μL). Deionized water was used as a reference. A water–water scan with four re-scans and a K-peptide–water scan with one re-scan in the range of 10–100 ${}^{{}^{\circ}}$C and a scanning speed of 1 ${}^{{}^{\circ}}$C·min${}^{-1}$ were collected. The K-peptide concentration was set to 2 mg·mL${}^{-1}$ for a satisfactory signal intensity with relatively low signal-to-noise ratio.

### 2.3. DFT Calculations

**Scheme 1:**
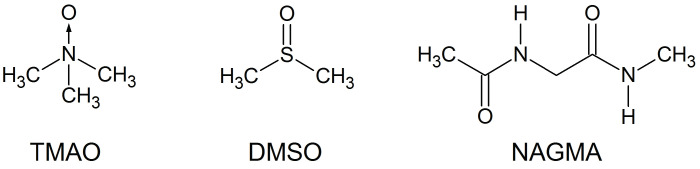
Chemical structures of molecules used for DFT calculations.

All quantum mechanical calculations were performed with the help of supercomputers of the Academic Computer Center (TASK, Gdańsk, Poland) with Gaussian 09.revD01 software [35]. Final structures were optimized at the B3LYP/6-311++G(d,p) level of theory [36,37,38], including the CPCM solvent model [39,40] and the GD-BJ3 dispersion correction [41] at the final calculation steps. The selected DFT method is optimal for small organic molecules, while the selected basis set provides a satisfying accuracy of calculation and a good agreement with real-life systems. All of the preparatory structures (see Scheme 1) were prepared, and the visualization of results were performed with the Avogadro v.1.2.0 software (Kitware, Inc., Clifton Park, NY, USA) [42].

The calculations were performed according to the following procedure, which is similar to that presented in our previous paper [43]: (1) the raw structures of all system components (TMAO, DMSO, and NAGMA) were first optimized in vacuo, and then, the results of this preparatory step were optimized within the CPCM solvent model; (2) the water molecules were added to these optimized structures close to various interaction sites, and then, each system was optimized again; and (3) the optimized complexes exhibiting the lowest energies were combined in all possible variations and configurations to form larger systems of NAGMA, water, and TMAO or DMSO and were optimized again. None of the resulting complex structures exhibited negative vibration frequencies.

Based on the results of the theoretical calculations, the cause of specific shifts and intensity changes in the experimental IR of the complexes were identified. In this paper, we focused mainly on the possibility of the formation of direct or indirect interactions between NAGMA and TMAO or DMSO.

## 3. Results and Discussion

### 3.1. Does the K-Peptide Have Any Structure in a Solution?

Before any interaction studies could be performed, an additional experimental step had to be performed for the K-peptide. Theoretically, without any a priori knowledge, we can assume that in aqueous solutions, this short peptide has a few different structures. These structures can be divided into two main groups: unstructured (or unfolded) free peptide, and structured (or organized) peptide or its clusters. The interpretation of other experimental and computational results depends on the characteristics of the structure; thus, using two experimental methods, we had to determine the structural type first.

**Figure 1:**
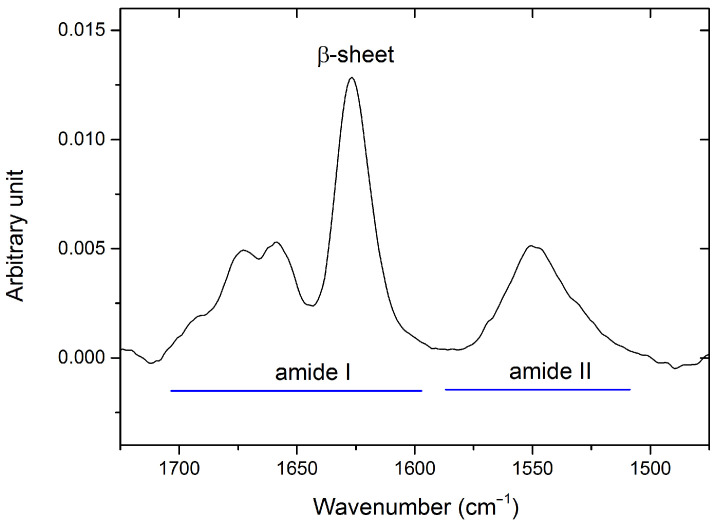
The amide I and II regions of the ATR-FTIR spectrum of the K-peptide in solution. The predominant β-sheet band (ca. 1625 cm${}^{-1}$) is marked.

ATR-FTIR spectroscopy of the aqueous protein sample gives information about the secondary structures present in a peptide solution. First, the K-peptide was re-suspended in deionized water to a final concentration of 40 mg·mL${}^{-1}$, and its ATR-FTIR spectrum was measured. The amide I band of the K-peptide was uncovered by subtracting the pure water spectrum (see Figure 1). The shape of the band indicates that β-sheets are its main component: the most predominant sub-band in the whole region is the β-sheet indicator band at ca. 1625 cm${}^{-1}$. Usually, the band shape of the amide I region of unstructured peptides exhibits a broad sub-band with a maximum at ca. 1650 cm${}^{-1}$. This sub-band is not present in the K-peptide spectrum; thus, we provide evidence that the peptide is folded in water. However, as the K-peptide is assumed to form β-amyloid structures, the folded form can be polydispersed. The other experiments and calculations were not affected by these results, but knowing what kind of structure is present in the solutions is necessary.

**Figure 2:**
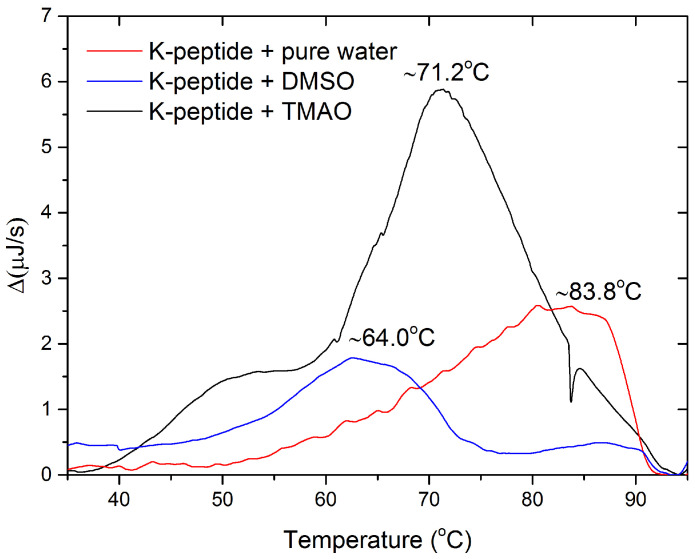
Raw thermograms of the K-peptide in different solutions. The maximum of each graph is marked with an appropriate temperature.

Differential scanning calorimetry (DSC) provides this knowledge of the types of structures present in the solutions. The K-peptide suspension (1.0 mL of 2 mg·mL${}^{-1}$) was placed in one cell of the calorimeter, while pure water was placed in the reference cell. The resulting thermogram is presented in Figure 2 (red line). We decided not to convert the raw thermograms to the molar Cp(T) because the exact concentration and molar mass of the peptide aggregate were uncertain. The presented graphs were calculated as the differences between the thermograms corresponding to the peptide’s solution and the pure water, DMSO, or TMAO solution; hence, the Δ sign in the μJ/s axis. Only a straight line was used for the baseline correction because the characteristics of each transition did not allow us to use the more common sigmoidal (or “chemical”) function. However, such raw thermograms provided sufficient information about K-peptide denaturation, and a conclusion could be drawn. The thermogram exhibited a characteristic broad denaturation transition to polydispersed polymers. The abrupt ending of the band at ca. 85 ${}^{{}^{\circ}}$C for the K-peptide in pure water indicates that the slow denaturation process is terminated by an irreversible denaturation step (a re-scan confirmed this observation; data not shown). The exact denaturation would have higher transition temperature than the maximum 100 ${}^{{}^{\circ}}$C of the measurement, as indicated by the rising slope of the thermogram. In the case of a more standard single peptide or protein molecules with well-defined structures, the recorded bands are much narrower, while amorphous aggregates exhibit a complex set of transitions [44]. The K-peptide cannot be clearly classified into any of the above-mentioned categories, and the hypothesis that the K-peptide forms polydispersed β-amyloid structures is correct.

### 3.2. FTIR

The spectra isolation method used in this work allows us to extract information on possible small changes in the shape of a given solution component. With a fixed osmolyte concentration, only changes caused by the presence of the K-peptide can be detected. However, the amount of water in a sample also changes because the volume of the peptide imposes a lower number of water molecules in the solution if the osmolyte’s concentration is fixed. Thus, small changes due to hydration/dehydration of the osmolyte may occur in such situations and must be taken into account. Without this procedure, all changes in the solution spectra could be falsely ascribed to osmolyte–K-peptide interactions. The easiest way to verify if the observed band shape is caused by such a phenomenon is to measure a series of osmolyte’s solutions’ spectra in the same range, where each spectrum corresponds to a slightly different osmolyte’s concentration. A changeability (or excess) spectrum can be easily calculated from such a series and compared with the one corresponding to the osmolyte–K-peptide system.

**Figure 3:**
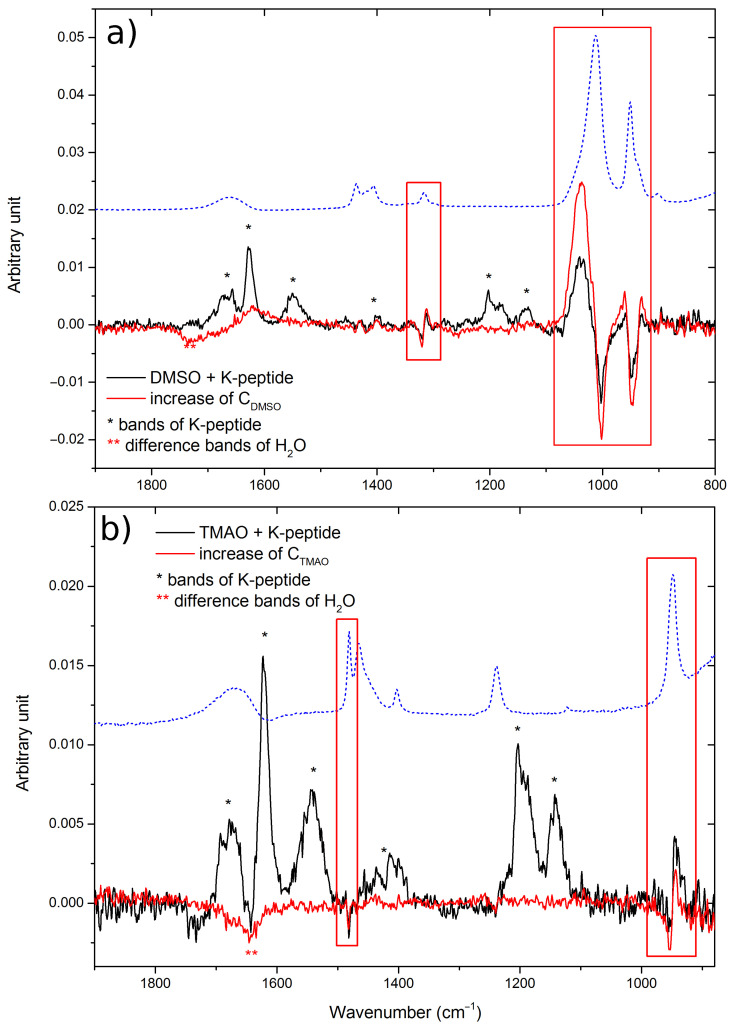
Isolated changeability spectra of DMSO (**a**) and TMAO (**b**) systems corresponding to possible interactions in aqueous solutions. Blue dashed lines correspond to pure component spectra. The most important differential bands and corresponding bands in ordinary spectra are marked with rectangles. Concentrations of TMAO or DMSO in the case of red graphs varied to reflect changes in the molar absorption caused solely by changes in the the amount of water available. The concentrations of these compounds, presented as black graphs, was fixed to obtain only the parts of the spectra that correspond to changes caused by the presence of the K-peptide. Band regions ascribed to the K-peptide and water are marked with asterisks. The intensities of all graphs were adjusted for easy comparison of the changes and to fit the panel.

DMSO and TMAO virtually do not directly interact with the K-peptide in aqueous solutions. Although changeability spectra are not flat and some difference bands are present, all of these changes in band shape can be easily attributed to the change in the osmolyte–water concentration ratio. All major shifts in the osmolyte bands (marked with red boxes) are exactly the same in the case of both the osmolyte–water–K-peptide and osmolyte–water systems (Figure 3). Some additional bands, marked with asterisks, can be easily ascribed to the K-peptide self-bands or changes in the water OH-bending band. Although the molar concentration of osmolyte in the system with the K-peptide is constant, the presence of the peptide causes dehydration of the osmolyte. In other words, a simple competition between DMSO or TMAO and the K-peptide occurs for the water molecules. The osmolyte is hydrated to a smaller extent when the peptide is present in the solution, and the same is true for the peptide.

**Figure 4:**
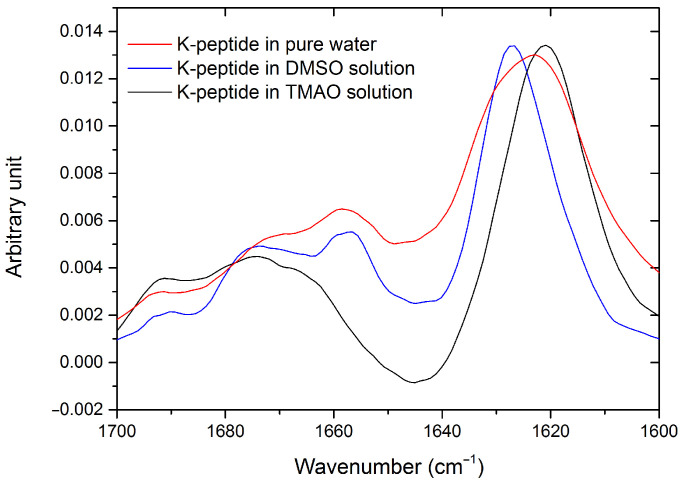
The amide I region of the K-peptide ATR-FTIR spectra in pure water and in the presence of TMAO or DMSO. All spectra correspond to the highest available concentrations of the K-peptide, and TMAO or DMSO.

Concluding whether such a phenomenon is favorable or unfavorable from the peptide stability point of view is difficult. However, a comparison of the K-peptide amide I bands in various solutions (see Figure 4) clearly indicates that C=O bonds are weaker when compared with the pure water solution in the case of TMAO (shifts to lower wavenumbers, ca. 1621 cm${}^{-1}$) and stronger in DMSO solutions (shift to higher wavenumbers, ca. 1627 cm${}^{-1}$). Stronger C=O bonds translate to weaker hydrogen bonds in the β-sheet structure. Thus, the β-sheet structure, based mainly on intramolecular hydrogen bonds, appears to be stronger in the solution of TMAO. Moreover, the shape of the amide band in a water solution can be considered a superposition of these two populations of C=O bond types, as the the wider β-sheet band is clearly composed of two sub-bands, corresponding roughly to well-defined single β-sheet peaks in DMSO and TMAO solutions. As no direct interactions are formed in solutions of both osmolytes, these changes in the shape of the amide I band of the K-peptide can be ascribed to modifications of its nearest solvent neighborhood.

### 3.3. DFT

**Figure 5:**
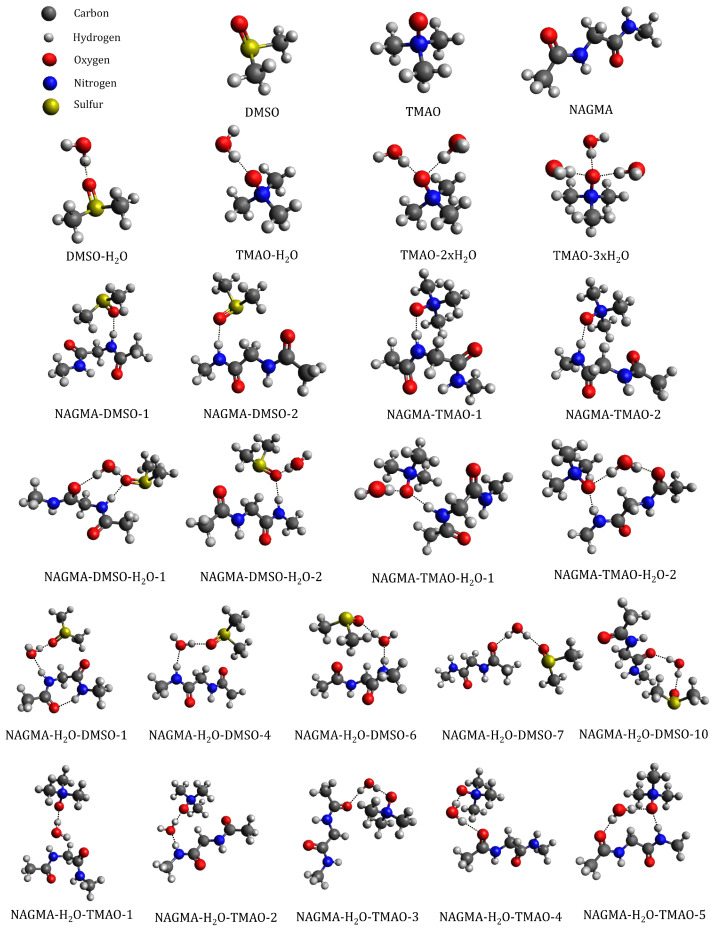
Optimized structures of DMSO, TMAO, NAGMA, and their complexes. The names of these structures are the same as in Table 1.

The DFT calculations gave insights into the possible interactions between various mixture components. The selected optimized structures of the complexes from Table 1 are presented in Figure 5. Although the systems were minimal (NAGMA instead of a long polypeptide chain, the CPCM model, and an additional water molecule), the IR results are in good agreement with general observations drawn from the theoretical calculations. In our previous papers, such systems reflected the changes in the band shape of the OD band well in similar complex solutions [33,45].

An isolated DMSO molecule exhibited a high-intensity S=O stretching band at 942 cm${}^{-1}$, and any interaction weakened this bond and shifted its position to higher wavenumbers (941.93 ± 4.1 cm${}^{-1}$, based on 12 structures). If the shift originated in the DMSO–water or DMSO–NAGMA, interaction was irrelevant. However, in one significant outlier structure, NAGMA-DMSO-H2O-2, 922.86 cm${}^{-1}$, the S=O bond was almost perpendicular to the N–H bond of NAGMA, i.e., the DMSO surface was closer to the surface of NAGMA in contrast with other optimized structures, where the angle between these two bonds was always larger than 90${}^{{}^{\circ}}$. Such a large shift, indicating a direct DMSO–NAGMA interaction, should be visible in the FTIR spectrum; however, all changes within the range of the S=O band are virtually identical for the DMSO–water and DMSO–water–NAGMA systems. Thus, we can conclude that direct interactions between DMSO and NAGMA are so rare that detecting them with a conventional FTIR setup is impossible.

TMAO-based systems act differently. The addition of water to an isolated TMAO molecule causes a shift in the N–O stretching band, ca. 935 cm${}^{-1}$, toward higher wavenumbers, which indicates that the N–O bonds become stronger when TMAO and water interact (a shift to ca. 940–945 cm${}^{-1}$). The almost single N–O bond in this case takes on the nature of a double bond. The analogous bond in the DMSO molecule is stronger in the isolated molecule, and an interaction does not exert such a strong impact on its quality, as seen in the FTIR spectra and DFT calculation results.

Although the behavior of the N–O bond in different environments seems illogical, this behavior is well reflected in the shifts predicted by the DFT calculations. If the tri- or di-hydrated TMAO is taken as a reference structure, a TMAO–NAGMA interaction causes the N–O band to shift to lower wavenumbers. The results of the DFT calculations do not exclude the possibility of close TMAO–water–peptide interactions. However, such a direct interaction should be accompanied with the formation of an additional band originating in a different frequency than the N–O bond, which interacts with water molecules of the K-peptide hydration shell (a split of the N–O band). This formation is not observed in the experimental spectra; thus, we conclude that such interactions are non-existent. Such a conclusion stays in good agreement with our previous experimental investigation of doubly affected water in systems of TMAO and model dipeptides [31]. In light of the MD simulation from that paper, the better hydration of the N–O group (i.e., an effect similar to dissolution) can be caused by preferential hydrophobic TMAO–peptide interactions: the N–O group is preferentially oriented toward bulk water. Although this interaction cannot be verified with our scheme of DFT calculations, the preferential hydration of TMAO and the accompanying dehydration of the peptide are well reflected in the DSC results.

### 3.4. DSC Results

As mentioned earlier, the K-peptide suspended in water is fairly stable, and the slow denaturation transition is terminated at very high temperatures (presumably higher than 90 ${}^{{}^{\circ}}$C) by irreversible denaturation. Any addition to the solution (DMSO or TMAO) significantly lowers the denaturation temperature and changes the overall characteristics of the transition.

TMAO is generally considered a good protein stabilizer. However, as seen in our previous paper, TMAO promotes the strong thermal irreversible aggregation of some proteins. The K-peptide is no different, and the thermogram obtained for the TMAO–peptide system reflects this fact very well. The initial slow denaturation process of the peptide complex structure is completely changed when TMAO is present in a solution. First, TMAO makes solvating the peptide difficult, as most K-peptides do not re-suspend well in the presence of an osmolyte. The supernatant, which was collected for the DSC experiment, had a significantly lower peptide concentration, yet the enthalpy of denaturation was significantly higher than that for the fully re-suspended peptide in pure water. The maximum of the denaturation peak was also lowered and accompanied by a strong irreversible transition (no peak was observed in the re-scans). The exact structure of the K-peptide in a solution is not known; however, poor hydration in the presence of TMAO, as we concluded on the basis of FTIR and DFT results, significantly destabilizes it thermally in contrast with the pure K-peptide solution. Moreover, the pre-transition peak (about 50 ${}^{{}^{\circ}}$C) may indicate that, at this temperature, some suspended peptide aggregates are broken down into smaller fragments, which are then highly destabilized by an unfavorable solvent properties, modified by the presence of the osmolyte. Hence, the denaturation involves high peptide–water structure reorganization and a significantly higher enthalpy of denaturation is observed, despite the fact that the concentration of the K-peptide is smaller than in the case of pure water and DMSO solutions.

DMSO, on the other hand, exhibits slightly more destabilizing properties than TMAO in terms of denaturation temperature. Although the solubility of the K-peptide is much higher than in the case of other two kinds of solution, the enthalpy of transition is the lowest, and the asymmetry of the peak indicates an irreversibility of the process (confirmed with the lack of any peaks in the re-scan).

## 4. Conclusions

In light of our experimental and theoretical results, we conclude that both DMSO and TMAO do not interact directly with the K-peptide in an aqueous solution. Although some changes in the vibrational structure of both osmolytes are visible in the FTIR spectra, when the peptide is present in their solutions, they can be ascribed solely to the effect of a concentration change. All FTIR spectra changes can be explained in term of simple solution mixing when co-solutes (i.e., the osmolytes and the peptide) do not interact with each other, and at least at the level of intermolecular interactions, both osmolytes stay neutral for the K-peptide.

Both osmolytes, DMSO and TMAO, favor diluted solutions and prefer interactions with water molecules. However, in both cases, the mechanism of this preference is different. DMSO hydration is hydrophobic in nature [30], yet this molecule regains some rotational freedom in a fully closed hydration cage, and its hydration is favorable from an entropic point of view. In more concentrated solutions, a not fully developed hydration layer is held down by favorable weak interactions. Thus, the decrease in available water in its surrounding, caused by the addition of the K-peptide, forces the DMSO molecule to hold to all available water without any formation of new interactions with the peptide or its aggregates surface. The hydration of TMAO is different from that of DMSO, and the most important forces driving those interactions are enthalpic in nature [31]. Very strong interactions with water and a relatively high number per TMAO molecule (even three H_2_O⋯O interactions) are not easily exchanged for one weaker interaction with the protein surface. Such an interaction would also involve the release of other water molecules from the TMAO and peptide surface to the bulk. Our recent molecular dynamics simulations of TMAO and dipeptide systems confirm such an explanation of the observed effects [31]. In those studies, a TMAO molecule was oriented toward the model dipeptide surface with its hydrophobic part, enhancing the hydration layer of the peptide, and avoided any direct contact, maintaining its strongly bounded water molecules. These two different mechanisms, or two different modes of competition for water in osmolyte–peptide solutions, are not directly noticeable in the shape of the osmolytes’ spectra; however, the possibility of such indirect interactions are reflected in the shape of the amide I band of the K-peptide and their influence on thermal stability of K-peptide aggregates. The presumed peptide dehydration in a TMAO solution may be beneficial at first, as indicated by the position of the β-sheet band; however, this dehydration may promote aggregation and the general irreversibility of the denaturation processes at higher temperatures, as seen in the DSC thermograms.

## Figures and Tables

**Table 1:** The position of stretching vibrations of S=O and N–O bonds of DMSO and TMAO, respectively, obtained for optimized structures of aqueous complexes with the NAGMA peptide model. The structures were selected according to the lowest energy criterion (energies calculated with the B3LYP/6-311++G(d,p) level of theory).

Structure	$\nu_{S=O}^{1}$/cm${}^{-1}$	$\nu_{S=O}^{2}$/cm${}^{-1}$
DMSO	941.93	1009.35
DMSO-H2O	931.11	1011.53
NAGMA-DMSO-1	932.07	1015.58
NAGMA-DMSO-2	934.79	1009.37
NAGMA-DMSO-H2O-1	931.74	1010.46
NAGMA-DMSO-H2O-2	922.86	1010.81
NAGMA-H2O-DMSO-1	929.71	1008.20
NAGMA-H2O-DMSO-4	930.80	1009.07
NAGMA-H2O-DMSO-6	927.57	1015.93
NAGMA-H2O-DMSO-7	936.52	1007.59
NAGMA-H2O-DMSO-10	932.60	1012.42
**Structure**	$\nu_{N-O}^{1}$ **/cm** ${}^{-1}$	$\nu_{N-O}^{2}$ **/cm** ${}^{-1}$
TMAO	935.37	—
TMAO-H2O	946.20	—
TMAO-2xH2O	951.66	—
TMAO-3xH2O	955.62	—
NAGMA-TMAO-1	942.06	—
NAGMA-TAMO-2	943.53	—
NAGMA-TMAO-H2O-1	952.66	—
NAGMA-TMAO-H2O-2	950.02	—
NAGMA-H2O-TMAO-1	940.77	947.86
NAGMA-H2O-TMAO-2	949.48	950.68
NAGMA-H2O-TMAO-3	941.65	947.13
NAGMA-H2O-TMAO-4	942.02	948.17
NAGMA-H2O-TMAO-5	953.49	—

## Data Availability

All of the data can be made available upon request.

## Abbreviations

The following abbreviations are used in this manuscript:

AA	Amino acid
ATR	Attenuated total reflectance
CPCM	Conductor-like polarizable continuum model
DFT	Density functional theory
DMSO	Dimethyl sulfoxide
DSC	Differential scanning calorimetry
FTIR	Fourier-transform infrared spectroscopy
NAGMA	*N*-acetyl-glycine-methylamide
TMAO	Trimethylamine *N*-oxide
